# Clinical outcome and patient satisfaction using biodegradable (NasoPore) and non-biodegradable packing, a double-blind, prospective, randomized study^☆^

**DOI:** 10.1016/j.bjorl.2016.01.001

**Published:** 2016-03-28

**Authors:** Pawel Krzysztof Burduk, Malgorzata Wierzchowska, Blazej Grześkowiak, Wojciech Kaźmierczak, Katarzyna Wawrzyniak

**Affiliations:** a Nicolaus Copernicus University, Faculty of Medicine, Otolaryngology and Laryngological Oncology Collegium Medicum, Toruń, Poland; b University Hospital, Department of Otolaryngology and Laryngological Oncology, Bydgoszcz, Poland; c Nicolaus Copernicus University, Faculty of Medicine, Department of Pathophysiology of Hearing and Balance System, Toruń, Poland; d Nicolaus Copernicus University, Faculty of Medicine, Department of Anesthesiology and Intensive Therapy Collegium Medicum, Toruń, Polônia

**Keywords:** FESS surgery, Biodegradable packing, Patient satisfaction, Mucosal healing, Follow-up, Cirurgia FESS, Tampão biodegradável, Satisfação do paciente, Cicatrização da mucosa, Seguimento

## Abstract

**Introduction:**

Nasal packing after endoscopic sinus surgery is used as a standard procedure. The optimum solution to minimize or eliminate all disadvantages of this procedure may be accomplished using biodegradable packs.

**Objective:**

The aim of this study was to compare patient satisfaction and clinical outcome associated with absorbable and non-absorbable packing after FESS.

**Methods:**

In total, 50 patients were included in a prospective, double-blind, randomized trial. One side was packed with polyurethane foam, while the opposite side was packed with gauze packing. On the 2nd, 10th, and 30th postoperative day, the patients were questioned with the aid of a visual analog scale. The standardized questionnaires for bleeding, nasal breathing, feeling of pressure, and headache were used. The presence of synechiae, infection, or granulation was noted and recorded with the video-endoscopy.

**Results:**

A significant difference according to lower pressure was found in the NasoPore group compared to the controls on day ten after surgery. The NasoPore packing had lower scores with respect to postoperative nose blockage on the 2nd and 10th days. Mucosal healing was better for the NasoPore group, both at day ten and 30 compared with the control group.

**Conclusion:**

The overall patient comfort is higher when using NasoPore compared to non-resorbable traditional impregnated gauze packing. Intensive saline douches applied three to four times per day are mandatory after the operation to prevent synechiae formation and fluid resorption by the packing.

## Resumo

**Introdução:**

O tamponamento nasal após cirurgia sinusal endoscópica é procedimento de rotina. A solução ideal para minimizar ou eliminar as desvantagens desse procedimento pode ser alcançada com o uso de tampões biodegradáveis.

**Objetivo:**

O objetivo deste estudo foi comparar a satisfação do paciente e o desfecho clínico associados ao uso de tampões absorvíveis e não absorvíveis após a cirurgia funcional dos seios paranasais (*FESS*- *Functional Endoscopic Sinus Surgery*).

**Método:**

No total, 50 pacientes foram incluídos neste estudo prospectivo, duplo-cego e randomizado. Um dos lados foi tamponado espuma de poliuretano, enquanto no outro lado foi realizado um tamponamento com gaze. Nos 2°, 10° e 30° dias após a operação, os pacientes foram questionados com a ajuda de uma escala analógica visual. Foram empregados questionários padronizados para sangramento, respiração nasal, sensação de pressão e cefaléia. A presença de sinequias, infecção ou granulação foi registrada por videoendoscopia.

**Resultados:**

Foi observada diferença significante, da sensação de pressão, tendo sido menor no lado tratado com NasoPore *vs.* controles no 10° dia após a cirurgia. O tamponamento com NasoPore obteve escores mais baixos com respeito ao bloqueio nasal pós-operatório no 2° e 10° dias. A cicatrização da mucosa foi melhor no lado do NasoPore, mas no 10° e 30° dias, os resultados foram comparáveis aos do lado de controle.

**Conclusão:**

O conforto geral do paciente é maior com o uso de NasoPore *vs.* tamponamento tradicional com gaze besuntada não reabsorvível. O uso vigoroso de jatos de solução de salina aplicadas 3-4 vezes ao dia é um procedimento obrigatório após a cirurgia, para evitar a formação de sinequias e para uma absorção natural do tampão.

## Introduction

Chronic rhinosinusitis is a very common disease, and the success of treatment is dependent on effective surgery and postoperative care. Endoscopic sinus surgery (ESS) has become the gold standard for the treatment of inflammatory, benign and selected malignant pathology.^1^, ^2^ The main principles are re-establishing ventilation and drainage without scarring, synechiae, and obstruction.^3^, ^4^, ^5^ To achieve these results, the middle meatus is often packed. This procedure should stabilize the middle turbinate, prevent synechiae formation, and act as a hemostatic agent.^3^, ^4^, ^5^, ^6^, ^7^ However, nasal packing could be a source of pain, nasal obstruction, bleeding, and discomfort during pack removal.^5^, ^6^ These disadvantages are mostly compared with non-absorbable nasal packing.^3^, ^5^, ^6^, ^7^, ^8^, ^9^

Recently, different absorbable biomaterials have become available for use as middle meatus packing after functional endoscopic sinus surgery (FESS).^3^, ^4^, ^5^, ^6^, ^7^, ^8^, ^10^ These kind of packs do not need to be removed and therefore improve patient comfort after surgery.^3^, ^4^, ^7^, ^8^, ^10^ The material prevents synechiae formation and stabilizes the middle turbinate. It starts to dissolve within a few days and can be washed out or suctioned from the nose.^5^, ^8^, ^10^

NasoPore (Polyganics – Groningen, The Netherlands) is a biodegradable synthetic polyurethane foam, which was used in the current work. The polyurethane bonds provide strong initial compressive mechanical properties, whereas the hydrophilic component takes-up the water or blood and is gradually fragmented. The aim of this study was to compare patient satisfaction and clinical outcome associated with the absorbable and non-absorbable packing after FESS.

## Methods

### Study design

A prospective, double-blind, randomized trial; one side was packed with polyurethane foam after bilateral sinus surgery while the opposite side was filled with packing composed of traditional impregnated gauze strip. This study was approved by the Bioethics Committee of the Nicolaus Copernicus University (KB 326/2013) and written informed consent was obtained from all participants.

A total of 50 patients were included from the Dep. of Otolaryngology and Laryngological Oncology. The mean age was 47.5 years (±9.8); 22 female and 28 male patients were included. The inclusion criteria were chronic rhinosinusitis (CRS) with or without nasal polyps according to the EPOS guidelines^11^ and symmetrical pathology between the nasal cavities based on computed tomography (CT) scan.^12^ The study included 38 patients with CRS with nasal polyps and 12 without nasal polyps. The exclusion criteria were septoplasty, turbinate surgery, or known intolerance to polyurethane. In each case, bilateral surgery was performed to the same extent. The research was approved by the local ethics committee and informed consent was obtained before the study. The patients were computer-randomized to packing the right or left side with NasoPore and the other side with gauze strip. In all cases, the packing was placed in the middle meatus at the end of the surgery.

### Surgery

**Figure 1:**
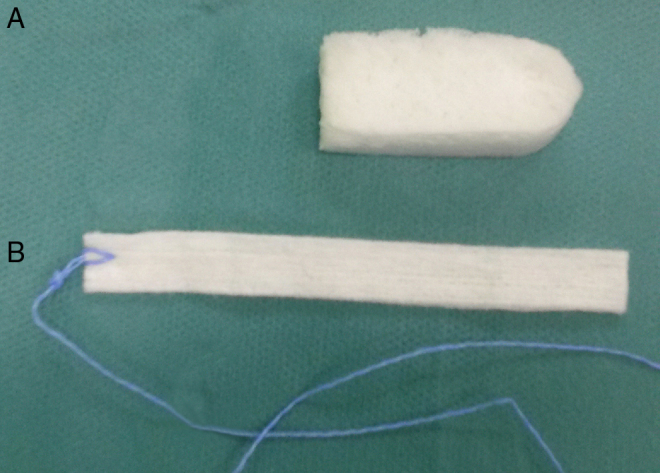
NasoPore (A) and gauze strip (B) packing.

The surgery was performed under general anesthesia by one surgeon. To minimize bleeding and optimize the surgical field, the procedure used premedication with clonidine and total intravenous anesthesia (TIVA), as described previously.^13^ Preoperatively, all of the patients received intravenous antibiotics (cefuroxime 1.5 g). At the end of the surgery, the surgeon was informed by the nurse of which side to use the NasoPore, which was randomly assigned. The opposite side was packed with non-resorbable gauze strip pack. Standard 4 cm NasoPore and 4 cm long gauze strip with an ointment (Fig. 1) were used. The procedure utilized 2 g Oxycort ointment (1 g contains 310 mg of hydrocortisone and 30 mg of oxytetracycline, as well as the base – Jelfa, Poland). The patients and the observer were not informed of which side had received the NasoPore or gauze packing.

### Follow-up

On the 2nd, 10th, and 30th postoperative day, a physician other than the operating surgeon questioned the patients with the aid of a visual analog scale (VAS) and performed nasal endoscopy. The non-absorbable packing was removed on the 10th day after surgery. The data collection was analogous to comparable studies, using standardized questionnaires for each side for the following parameters: bleeding, nasal breathing, feeling of pressure, and headache.^5^, ^6^, ^10^, ^14^ The parameters were determined using a VAS with possible values ranging from 0 (no symptoms) to 10 (maximum symptoms). The presence of synechiae, infection, granulation, or re-epithelialization was noted and recorded with the video-endoscopy on both sides on the 10th and 30th day after surgery. After discharge, all patients used an antibiotic (clarithromycin, 1000 mg daily for 10 days, nasal steroids (fluticasone furoate) once daily, and nasal saline douches up to three to four times daily).

### Statistical analysis

Statistical analysis was performed with Statistica software, v. 10. (StatSoft Inc.) The parameters were compared using the Wilcoxon signed rank test, McNemara test, and Shapiro–Wilk's test. The level of significance was defined as *p* < 0.05. The study population was calculated for error inherent in a test result. The power analysis of the investigation group was 80%.

## Results

Fifty patients were randomized and 100 sinus cavities were treated. The absorbable packing was put in 27 right sides and 23 left sides of the nasal cavities. The non-absorbable packing was put accordingly in 23 right and 27 left sides of the nasal cavities. Forty-nine patients completed the study. This was due to one patient refusing to attend follow-up, as he felt well. The VAS results for pressure, nose blockage, headache, and nasal pain are shown in Table 1. A significant difference according to pressure was found between the NasoPore and control sides on day 10 after surgery (*p* < 0.04). The patients reported lower filling of nose pressure on the NasoPore side. No differences were observed on the 2nd and 30th days post-surgery. The NasoPore packing had lower scores with respect to postoperative nose blockage (4.26 *vs.* 4.73, *p* < 0.04) on the 2nd and 10th days (1.81 *vs.* 2.29, *p* < 0.02; Table 1). The results were significant. However, there was no significance on the 30th day (0.45 *vs*. 0.68, ns). Nevertheless, slightly lower scores for headache and nasal pain were recorded for the NasoPore group during the follow-up visits, but the results were not significant (Table 1).

**Table 1:** Results of visual analog scale questionnaire during the follow-up visits.

	Pressure			Nose blockage			Headache			Nasal pain		
	N	C	*p*	N	C	*p*	N	C	*p*	N	C	*p*
Day 2 *n* = 50	3.03 (±3.01)	3.26 (±2.93)	0.15	4.26 (±2.98)	4.73 (±2.79)	0.04	3.41 (±3.1)	3.48 (±2.91)	0.79	3.32 (±3.29)	3.14 (±2.99)	0.37
Day 10 *n* = 49	1.9 (±1.93)	2.16 (±1.99)	0.04	1.81 (±1.79)	2.59 (±1.85)	0.02	1.78 (±1.88)	2.06 (±2.01)	0.14	1.8 (±1.86)	2.08 (±2.14)	0.10
Day 30 *n* = 49	0.23 (±0.58)	0.45 (±1.22)	0.06	0.45 (±0.96)	0.68 (±1.68)	0.62	0.28 (±0.55)	0.4 (±0.78)	0.11	0.38 (±0.93)	0.4 (±0.78)	0.49

N, Nasopore; C, control; *n*, number of patients.

Assessment of bleeding on packing removal demonstrated no differences. Minimal bleeding without any future intervention was observed for one case in each group.

**Figure 2:**
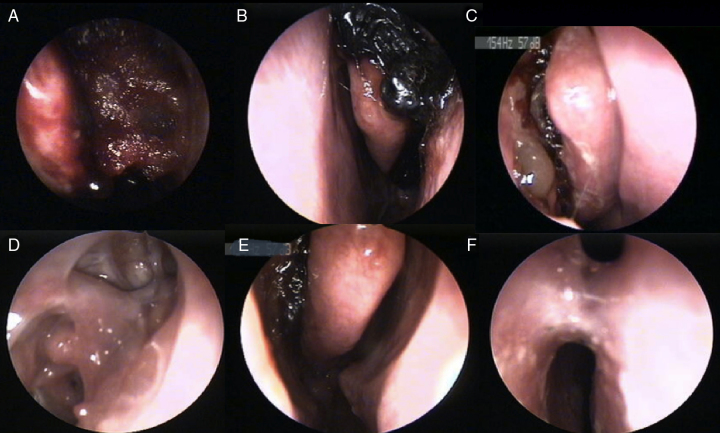
NasoPore at the end of surgery (A) and resorption process at 2nd, 10th, and 30th day (B–D), as well as some remnants of dressing after ten days post operation (E) and synechia formation (F).

Forty-nine subjects returned for the assessment of mucosal healing on the 10th and 30th days after operation. Endoscopic observations of wound healing after surgery revealed blood crusting, edematous swelling, and epithelialization. Mucosal healing (re-epithelialization) was better for the NasoPore group, both on day ten and 30, compared with the control group (*p* < 0.001, *p* < 0.06). At 10th day the re-epithalization in study group was 68.1% and reached over 95.7% at 30th day. The endoscopic view at 10th day was very satisfactory compared to control group, where only 32.7% of the operated field showed epithelization. In both groups at the 30th day, the re-epithelization level was over 90%, pointing to complete healing. Nevertheless, the re-epithelialization in the control group achieved a satisfactory level on the 30th day (90.2%). In this study, synechiae formation was observed in three of the NasoPore group and two of the control group (Fig. 2). No significance was observed (Table 2). In one case of non-absorbable packing, on the 30th day of follow-up, infection with mucopurulent discharge was found.

**Table 2:** Results of synechiae, infection, and re-epithelization between groups.

	Synechiae			Infection			Re-epithelization		
	N	C	*p*	N	C	*p*	N	C	*p*
Day 2 *n* = 50	–	–	–	–	–	–	–	–	–
Day 10 *n* = 49	0	0	ns	0	0	ns	68.1%	32.7%	<0.001
Day 30 *n* = 49	3	2	ns	0	1	ns	95.7%	90.2%	<0.06

N, Nasopore; C, Control; ns, not significant; *n*, number of patients.

Resorption of the absorbable packing was fluent in most cases (Fig. 2). In three cases, some remnants of the dressing on the 10th day were noted, which could result in synechiae formation in the late follow-up (Fig. 2). In these cases, the patients did not adhere to the recommendation of regular (three to four times per day) nasal douches.

## Discussion

The most important considerations after FESS operations are patient comfort, minimizing bleeding, reduction of discomfort associated with nasal packing, and proper mucosal healing. As the non-resorbable (removable) nasal packing can be very unpleasant, the different types of resorbable packing have been investigated.^4^, ^5^, ^6^, ^8^, ^10^ Some authors do not support the use of nasal packing at all.^6^, ^8^, ^10^, ^15^ Conversely, middle meatal packing has prevented lateralization of the middle turbinate, synechiae formation, and bleeding.^4^, ^6^, ^7^, ^10^ Using some absorbable materials, the mucosal healing process could be more effective and faster.^3^, ^4^ In some cases, the absorbable materials could also be associated with slower healing and synechiae formation. This is probably because of the possibility of osteogenesis initiation.^5^ One of the newly developed biodegradable nasal packing materials is the polyurethane foam NasoPore, which is used after FESS operations.^5^, ^10^ This material could also be impregnated with steroids or antibiotics to reduce the post-operative discomfort and achieve better clinical outcomes.^16^, ^17^

The aim of this study was to compare the efficacy of a biodegradable nasal packing (NasoPore) with a traditional gauze strip packing impregnated with ointment (2 g of oxytetracycline and hydrocortisone). The ointment was used to prevent the adherence of the packing to the mucosa.

The post-operative feeling of pressure was higher in the control group than on the NasoPore side. On post-operative days two and 30, this observation was not statistically significant. On day ten, the feeling of pressure was greater on the control side, resulting in a significant difference (*p* < 0.04). In the authors’ opinion, this was caused by resorption of the NasoPore, and by stable gauze strip packing and the formation of blood clots around the gauze material. Patient comfort appears to be improved by the resorbable packing. The same observations have been reported by other authors.^4^, ^5^, ^10^

Parameters including nose blockage, headache, and nasal pain were generally lower for the NasoPore group than the control group. On day ten, a statistically significant reduction of nose blockage (*p* < 0.02) in the NasoPore group was observed. This was caused by resorption of the packing with less debridement in the middle meatus compared to high secretion and edema mediated by the gauze packing on the other side. However, although there were no statistically significant differences for the observed parameters during the follow-up, patient comfort appeared to be much better in the NasoPore group. The same observations were made by other authors comparing the usage of different resorbable and non-resorbable packing materials.^3^, ^5^, ^6^, ^10^ The study has demonstrated that NasoPore does not significantly reduce the risk of post-operative bleeding. The same results were observed for other resorbable and non-resorbable nasal packing materials.^5^, ^6^, ^7^, ^9^, ^10^

The present study did not find any statistically significant differences between the packing materials used with regard to synechiae formation or infection.^5^, ^10^ Otherwise, if the patient did not respect the necessity of intense nasal rinsing in early post-operative period, the formation of synechiae would be highly likely. The partially dissolvable pack and all debridement should be suctioned out or washed out if remaining in place for longer than ten days. The remnants of the NasoPore could form a bridge between the middle turbinate and the lateral nasal wall as a point of synechiae formation.^8^ On the other hand, some absorbable materials or its remnants could be incorporated into regenerating mucosa or activate osteogenesis, leading to synechiae formation. This process is responsible for slower mucosal healing, as described by Shoman.^5^ The present study found a significantly better re-epithelialization process in the NasoPore group on day ten (*p* < 0.001) and nearly complete epithelialization 30 days after surgery (95.7%). It is thought that removing the non-absorbable packing could cause local mucosal bleeding and a prolonged phase of blood crusting, which delays epithelialization. Nevertheless, the difference almost disappeared by the late follow-up visit (*p* < 0.06). The same results were observed by Shoman.^5^

Overall, the NasoPore packing results in better patient comfort and a better healing process after FESS surgery.

## Conclusion

Using a resorbable NasoPore packing after FESS, the feeling of pressure and nose blockage in the early post-operative period were significantly reduced. The overall patient comfort was higher compared to non-resorbable traditional gauze strip packing. The wound healing was better when using NasoPore, but future investigations are required. Intensive saline douches, three to four times per day, are mandatory after the operation to prevent synechiae formation and fluid resorption by the packing.

## Conflicts of interest

The authors declare no conflicts of interest.
